# Three electron beams from a laser-plasma wakefield accelerator and the energy apportioning question

**DOI:** 10.1038/srep43910

**Published:** 2017-03-10

**Authors:** X. Yang, E. Brunetti, D. Reboredo Gil, G. H. Welsh, F. Y. Li, S. Cipiccia, B. Ersfeld, D. W. Grant, P. A. Grant, M. R. Islam, M. P. Tooley, G. Vieux, S. M. Wiggins, Z. M. Sheng, D. A. Jaroszynski

**Affiliations:** 1 SUPA, Department of Physics, University of Strathclyde, Glasgow, G4 0NG, UK; 2 Institute of Physics of the ASCR, ELI-Beamlines, Na Slovance 2, 182 21 Prague, Czech Republic; 3 Laboratory of Laser Plasmas and Department of Physics and Astronomy, Shanghai Jiao Tong University, Shanghai 200240, China

## Abstract

Laser-wakefield accelerators are compact devices capable of delivering ultra-short electron bunches with pC-level charge and MeV-GeV energy by exploiting the ultra-high electric fields arising from the interaction of intense laser pulses with plasma. We show experimentally and through numerical simulations that a high-energy electron beam is produced simultaneously with two stable lower-energy beams that are ejected in oblique and counter-propagating directions, typically carrying off 5–10% of the initial laser energy. A MeV, 10s nC oblique beam is ejected in a 30°–60° hollow cone, which is filled with more energetic electrons determined by the injection dynamics. A nC-level, 100s keV backward-directed beam is mainly produced at the leading edge of the plasma column. We discuss the apportioning of absorbed laser energy amongst the three beams. Knowledge of the distribution of laser energy and electron beam charge, which determine the overall efficiency, is important for various applications of laser-wakefield accelerators, including the development of staged high-energy accelerators.

A short intense laser pulse propagating in underdense plasma can produce, by charge separation, electric fields that are orders of magnitude higher than those available in conventional radio-frequency accelerators^1^. Progress in the understanding of the relevant physics^2^,^3^,^4^,^5^,^6^, and advances in high-power laser technologies, have enabled the development of laser-plasma wakefield accelerators (LWFAs), as compact devices capable of accelerating electrons to high energies over millimetre-scale lengths^7^,^8^,^9^,^10^. Recently, acceleration of electron beams up to 4.2 GeV was reported by focusing 300 TW laser pulses into a 9 cm plasma waveguide^11^. Bunches as short as 1fs have been measured^12^,^13^ and the use of LWFAs as compact radiation sources has been demonstrated in a broad spectral range from terahertz^14^ to x-rays^15^ and *γ*-rays^16^. LWFAs are also being considered as drivers for free-electron lasers^17^,^18^,^19^,^20^,^21^,^22^,^23^.

Current research has focused on the production of high-quality, high-energy bunches, which have only been achieved for tens of pC charge^24^, but there are applications such as non-destructive-testing, ultra-fast studies in condensed matter, radiolysis, isotope production or radiotherapy that require high-charge, low-energy electron beams^25^,^26^,^27^,^28^,^29^. Ionisation-assisted injection in high-Z or clustering gas targets can increase the charge of LWFAs to 100s pC for 10s–100s MeV energies^30^,^31^,^32^. However, little attention has been given to the intrinsic property of LWFAs to emit high-charge electron beams with MeV energies at wide angles^33^,^34^. Not only is a general interpretation of the underlying physics lacking, but it is not clear how the oblique ejections influence the overall efficiency of LWFAs. Here we draw attention to these aspects and present a full characterisation of such wide-angle low-energy electrons through experiments and numerical simulations, also reporting the observation of a 100 s keV, nC electron beam ejected in the backward direction. The two low-energy electron beams, in addition to the forward high-energy beam, are found to be very stable over a wide range of laser and plasma parameters. Typically, the oblique beam has 1–2 MeV energy and 10s nC charge, produced in a hollow cone of 30°–60° opening angle with respect to the laser direction. Under proper conditions, typically with the onset of injection, electrons of 5–10 MeV or higher energy and 100s pC charge can fill the cone to form a broad “halo” pattern, which is combined with the high-energy forward bunch^35^. These low-energy beams are appealing for applications such as single-shot imaging or dosimetry, because of their high-charge and inherent capability to induce fluorescence over a large area. Knowledge of their properties is also important for the LWFA studies, as they are shown to be closely related to the injection dynamics, which are critical to the production of high-energy beams. The oblique and backward beams can also produce large fluxes of bremsstrahlung radiation if not properly dumped, leading to undesirable X-ray radiation background. Another important concern is the large amount of energy carried off the plasma by these high-charge beams, despite their low energies per electron, which have consequences for high energy staged accelerators. Our simulations show that for a laser beam shrunk to a transverse radius of 5–10 μm through self-focusing, a substantial fraction of laser energy, as high as 5–10%, can be transferred to oblique and backward electrons in a short propagation distance of 0.5–1 mm. Thus, the energy apportioning issue raises additional concerns about the overall efficiency of LWFAs.

## Results

### Numerical simulations of side-electron emission

Before showing experimental results, we present numerical simulations to illustrate the dynamics of the side-electron emission. We start with a general overview of the mechanism leading to the ejection of electrons at wide angles. We then provide examples of angular distributions and energy spectra of oblique electron beams. We finally discuss the properties of oblique beams for a wide range of laser and plasma parameters.

**Figure 1:**
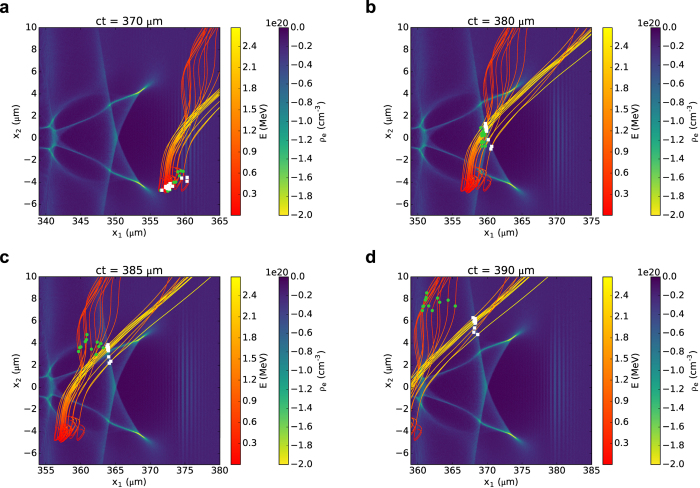
Snapshots of the electron density distribution produced behind an intense laser pulse propagating along the *x*_1_ direction in a plasma. Curves correspond to sample trajectories of electrons ejected at wide angles, with the line colour representing the electron energy. The symbols show the position at the given time of electrons streaming close (white squares) and further away (green circles) from the accelerating structure (bubble) interior.

When a short intense laser pulse travels in underdense plasma, its ponderomotive force pushes electrons away from high intensity regions, leaving approximately spherical ion cavities (“bubbles”) trailing behind the pulse^4^,^5^. Snapshots of the first two bubbles are shown in Fig. 1, obtained from two-dimensional particle-in-cell (2D PIC) simulations for a laser that is linearly polarised in the *x*_3_ direction and travelling along the *x*_1_ axis; details of the set-up are given in the Methods. The plots include typical trajectories of electrons ejected at wide angles, with their positions at different times, marked by symbols. Background electrons undergo longitudinal and transverse acceleration as they interact with the bubble fields, but most of them gain little energy and stream backwards relative to the bubble motion, mainly feeding the formation of further cavities^13^,^36^,^37^,^38^. On the other hand, electrons located off-axis at a distance close to the bubble radius form a dense sheath and experience strong accelerating fields (Fig. 1a). When reaching the bubble base (Fig. 1b), some of these can be trapped inside the bubble and accelerate to energies that can reach GeV levels. Most others, having insufficient longitudinal momenta, are not injected into the bubble, but eject from the plasma at an angle of 30°–60°, depending on the specific energy gain (Fig. 1c,d). For example, electrons (white squares in Fig. 1) streaming around the sheath of the first bubble gain more energy and are ejected closer to the laser propagation axis than those further away (green circles), which can also lose energy due to the decelerating field at the front of the second bubble. Electrons can also be injected into the second bubble, forming a hollow beam that is accelerated to nearly 300 MeV energies with 10 pC charge^39^. Here, however, we investigate the properties of electrons that are not trapped in the bubble and report an efficient acceleration mechanism for nC-level beams with 1–10 MeV energy.

**Figure 2:**
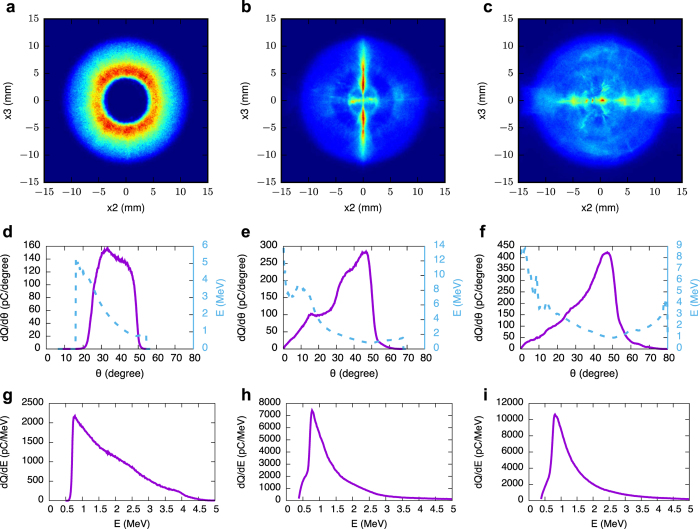
3D PIC simulations of spatial (**a–c**), angular (**d–f**) and spectral (**g–i**) distribution of oblique electrons on a screen 10 mm downstream from the plasma. The laser intensity and spot size are *a*_0_ = 2, *w*_0_ = 5 μm (left column), *a*_0_ = 2, *w*_0_ = 7 μm (middle column) and *a*_0_ = 3, *w*_0_ = 7 μm (right column). Dashed curves in Figures (**d–f**) show the energy dependence on angle. Further examples are given in the Supplementary Section.

In order to characterise the oblique beam quantitatively, we now consider 3D PIC simulations over a wide range of parameters for a laser linearly polarised in the *x*_2_ (horizontal) direction. The laser intensity *I*_0_ is expressed in terms of the normalised vector potential *a*_0_ = 8.5 × 10^−10^ *λ*_*L*_[μm](*I*_0_[W/cm^2^])^1/2^, with *λ*_*L*_ the laser wavelength. Here, *λ*_*L*_ = 0.8 μm. Results show that electrons are ejected over a wide cone with angles as large as 60° from the laser axis, and are often clustered in beamlets with properties that strongly depend on specific laser and plasma parameters. Representative examples are presented in Fig. 2 for electrons produced in a pre-ionised plasma with a density of 2 × 10^19^ cm^−3^, comparable with the parameter of experiments described below. Plots in the left column are obtained for *a*_0_ = 2, spot size *w*_0_ = 5 μm and propagation length 0.7 mm. In this case, injection into the bubble does not occur, and therefore no high-energy forward beams are formed. The bubble evolves little during propagation and the oblique electrons are ejected into a hollow cone with 36° mean angle, 7° rms divergence and 3.3 nC total charge. The mean energy is 1.7 MeV with 50% rms energy spread. The oblique beam carries a total energy of 2.8 mJ, corresponding to approximately 4% of the initial laser energy (68 mJ). If the spot size is increased to *w*_0_ = 7 μm (plots in the middle column), injection occurs after 0.2 mm propagation and ejected electrons fill in the cone, since they can gain a larger longitudinal momentum by entering the bubble. The mean angle is 33°, with 12° divergence, 7.4 nC total charge, 2.4 MeV mean energy and 135% energy spread. The total energy is 18 mJ, corresponding to ~13% of the initial laser energy (133 mJ). Also prominent in the plots are two narrow beamlets aligned along the vertical axis and having a mean angle of 26°, with 4.5 MeV mean energy, 90% energy spread and ~200 pC charge. This feature depends strongly on the thermal momentum distribution of the electrons before the arrival of the laser pulse, in particular along *x*_3_ (see Fig. 2c and S.1 and S.2 in the Supplementary Section). Electrons initially at rest cluster along the *x*_2_ axis, which is parallel to the laser polarisation direction, whereas an initial momentum spread causes the formation of patterns at different angles depending on the laser parameters. Particle tracking indicates that these beamlets correspond to short intense bursts around the time of injection and contain only a small fraction of the total charge in the cone, despite the high visibility in the plot. They are attributed to the increased contribution to the wake potential of the longitudinal variation of the electron density during laser evolution (self-focusing and compression) and charge build-up at the bubble base. The same conditions also favour the onset of injection^13^,^36^,^37^,^38^, explaining the link between ejection angle and injection dynamics, which leads to the cone filling in. Plots on the right column are obtained for *w*_0_ = 7 μm, *a*_0_ = 3 and propagation length 0.5 mm. Again electrons fill the entire cone, but cluster preferentially along *x*_2_. The mean angle is 38° with 13° divergence, 10 nC total charge, 1.8 MeV mean energy and 116% energy spread. The total energy is 19 mJ, corresponding to 6% of the initial laser energy (300 mJ). It should be noted that the fraction of laser energy driving the plasma wake depends on laser and plasma parameters. For example, about 30% of the initial laser energy is transferred to the plasma after 1 mm propagation for laser *a*_0_ = 3, spot size between 7 and 10 μm and plasma density of 5 × 10^18^ cm^−3^. On the other hand, for a plasma density of 1 × 10^19^ cm^−3^ the energy transfer is about 80% for the same laser conditions, and at higher densities the loss can be as high as 90% after 0.5 mm propagation.

As shown by the dashed curves in Fig. 2d–f, the electron energy is angle dependent, increasing from a mean value of about 1 MeV at large angles to almost 10 MeV on-axis. The associated energy spectra are presented in the bottom row of Fig. 2, showing typical energies of 1–2 MeV. Since electrons are continuously ejected during laser propagation, the bunch duration is typically picoseconds and depends on the interaction length. Simulations of neutral helium gas, where the ionisation process is included using the ADK model^40^, present similar beam properties (see Supplementary Section). In addition, the ejected electrons are also shown to produce strong magnetic fields as they cross the transverse plasma boundaries, in analogy to the flow of charged particles from an ionised to a non-ionised interstellar medium^41^,^42^.

**Figure 3:**
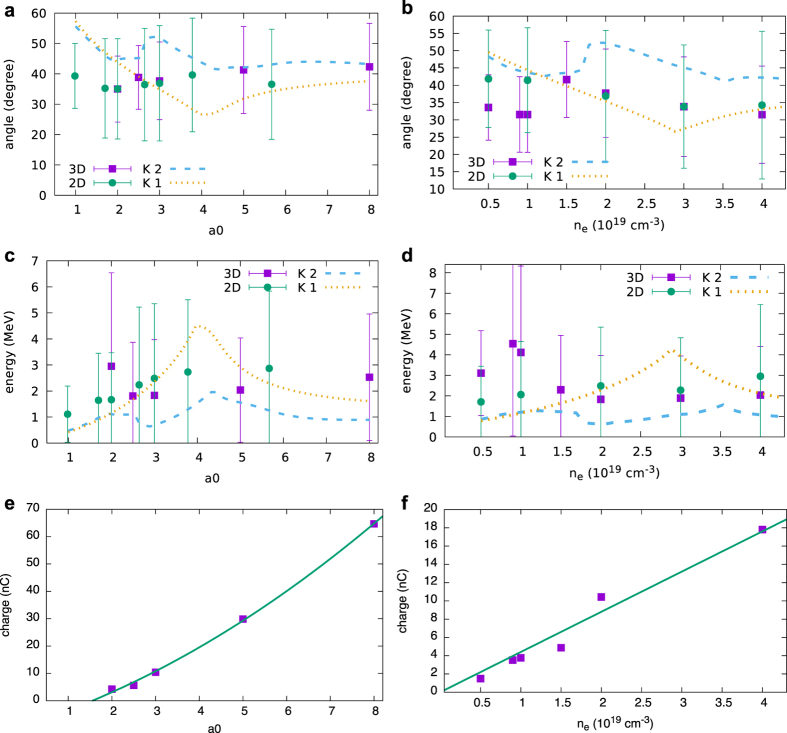
Dependence on laser *a*_0_ and plasma density of mean angle, energy and charge of oblique electrons from 3D (squares) and 2D (circles) simulations for spot size *w*_0_ = 7 μm and propagation length 0.5 mm. The error bars represent rms divergence and energy spread. (**a,c,e**) have been obtained for *n*_*e*_ = 2 ×10^19^ cm^−3^ and Figures (**b**,**d**,**f**) for *a*_0_ = 3. Curves in (**a–d**) show the mean energy and angle predicted by Kostyukov’s model^43^ for one (dotted line *K*_1_) and two (dashed line *K*_2_) bubbles. Curves in Figures (**e,f**) are quadratic and linear fits, respectively.

The properties of oblique beams for a wide range of laser and plasma parameters are presented in Fig. 3. The dependence of mean angle and energy on laser strength *a*_0_ are shown in Fig. 3a and c for plasma density 2 × 10^19^ cm^−3^, laser spot size *w*_0_ = 7 μm and 0.5 mm propagation length. At almost all the intensities, the ejected electrons fill the full 60° cone. There are no large changes in the energy and angle, since the non-linear laser evolution eventually causes the bubble to slow down, capping the energy gain (Figures S.5 and S.6 in the Supplementary Section). For the given range, the charge grows quadratically with *a*_0_ (Fig. 3e), which is expected if the laser expels a hollow column of plasma electrons with thickness determined by the bubble transverse size, which grows linearly with *a*_0_ (see Figures S.6 and S.7 in the Supplementary Section and the associated discussion). The dependence on plasma density is presented in Fig. 3b and d for *a*_0_ = 3, *w*_0_ = 7 μm and 0.5 mm propagation length. Again, beam properties change little, since the higher accelerating gradients achievable at high densities are countered by the shorter acceleration length and lower bubble speed (Figures S.5 and S.6 in the Supplementary Section). Higher energies are observed close to 1 × 10^19^ cm^−3^ due to short bursts of high energy electrons emitted during injection. At 5 × 10^18^ cm^−3^ there is no injection within the given propagation length, but injection occurs for laser spot size *w*_0_ = 10 μm, when a mean energy of 4.9 MeV is observed. At 1 × 10^18^ cm^−3^ there is no injection also for *w*_0_ = 10 μm and the mean energy is 1.8 MeV with 1 nC charge after 1 propagation. The charge increases linearly with density (Fig. 3f), as the volume of the hollow column of ejected electrons remains approximately constant (see Figure S.7 in the Supplementary Section and the associated discussion). Nevertheless, for low densities the accelerating structure evolves more slowly and electrons can be produced over longer distances. For example, the charge at 1 × 10^19^ cm^−3^ is 3.9 nC after 0.5 mm and 10.3 nC after 1 mm (Figure S.3 in the Supplementary Section).

The weak dependence of energy and angle on laser and plasma parameters is a consequence of the small range of momenta acquired by electrons not injected into the bubble. This is confirmed by comparing the above PIC simulation results with the predictions from Kostyukov’s semi-analytical reduced model^43^, where the bubble is modelled as a non-evolving ionic sphere moving in a plasma at a constant velocity and surrounded by a thin electron sheath. The trajectories of displaced background electrons have been calculated both for a single bubble and for two coalesced bubbles. Here, 50,000 sample electrons initially at rest and uniformly distributed are considered and the bubble radius and velocity are estimated from 3D PIC results (Figure S.6 in the Supplementary Section). The final beam properties are plotted in Fig. 3 and Figures S.4–S.5 in the Supplementary Section. Electrons initially close to the laser axis or located at a distance larger than the bubble radius gain little energy and are scattered at large angles or left unperturbed. Only electrons displaced by about the bubble radius are accelerated to MeV energies, capped by the onset of injection. Higher energies are possible for larger bubble sizes, and therefore longer acceleration lengths, but typically have exponential spectra, since the mean energy is still dominated by the bulk of less energetic electrons. Furthermore, the curves obtained for two attached bubbles indicate that the decelerating field at the front of the second bubble reduces the longitudinal energy gain and leads to larger ejection angle. A complete description of the process should include dynamic bubble evolutions (changes in size and velocity, density variations, asymmetries in shape and differences between successive buckets), which is only possible with 3D PIC simulations.

### Numerical simulations of backward electron emission

**Figure 4:**
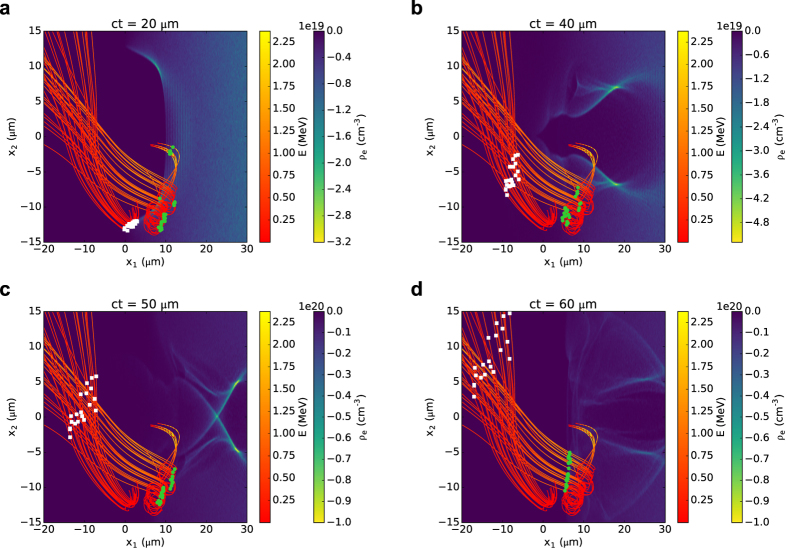
Snapshots of the electron density distribution at the left vacuum-plasma boundary *x*_1_ = 0 obtained from 2D PIC simulations for plasma density 2 × 10^19^ cm^−3^, laser spot size *w*_0_ = 7 μm and *a*_0_ = 5. Curves correspond to sample trajectories of backwards emitted electrons, with the line colour representing the electron energy. The symbols show the position at the given time of electrons originating from the plasma boundary (white squares) and from deeper inside the plasma (green circles).

Backward emission of low-energy electrons, opposite to the laser propagation direction, has also been observed, mostly during the initial stages of the laser-plasma interaction. Figure 4 shows sample trajectories obtained from 2D PIC simulations in the laboratory-frame for electrons located in the proximity of the vacuum boundary. The plasma starts at *x*_1_ = 0 with a 60 μm long linear up-ramp to a density of 2 × 10^19^ cm^−3^. Electrons close to the boundary (white squares) are ejected immediately on laser arrival, whereas electrons deeper inside the plasma (green circles) are accelerated and then ejected backwards by the plasma fields, with the formation of the bubble structure. Some electrons also stream back from further inside the plasma. The energy distribution of these electrons is exponential, with mean energy of 0.3–0.4 MeV, depending on laser intensity. Here only electrons with energies above 0.1 MeV have been considered. The ejection angle can reach 90°, but typically electrons form a 40°–50° cone around the laser axis. An accurate calculation of the charge requires 3D simulations in the laboratory frame over the full interaction distance, which are very computationally intensive. However, 3D simulations using a moving window indicate that charges of the order of 0.5 nC can be produced over the first 60 μm of interaction for *a*_0_ = 3, *n*_*e*_ = 2 × 10^19^ cm^−3^ and *w*_0_ = 7 μm, giving a total energy of the backward electrons of about 0.13 mJ. Simulations for different plasma densities show no significant change in mean energy and ejection angle, whereas the charge varies from about 0.15 nC at 1 × 10^19^ cm^−3^ to 0.8 nC at 4 × 10^19^ cm^−3^. The charge also depends on the laser *a*_0_, obtaining 1.8 nC with a mean energy of 0.4 MeV for *a*_0_ = 5, *n*_*e*_ = 2 × 10^19^ cm^−3^ and *w*_0_ = 7 μm. In contrast, for *a*_0_ = 2 the charge is 0.1 nC with a mean energy of 0.3 MeV.

## Experimental results

**Figure 5:**
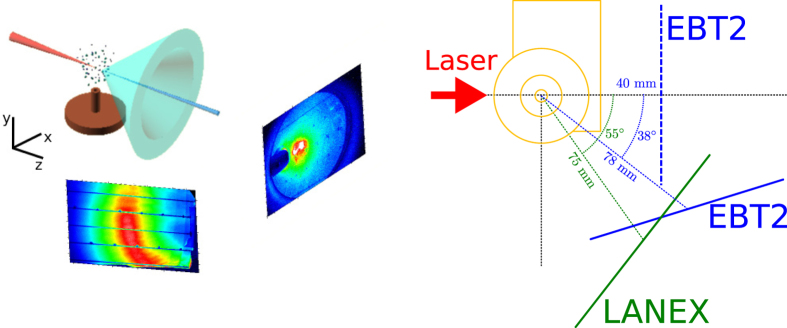
Schematic diagram of the experimental set-up showing the position of the main diagnostics used to characterise wide-angle electrons.

**Figure 6:**
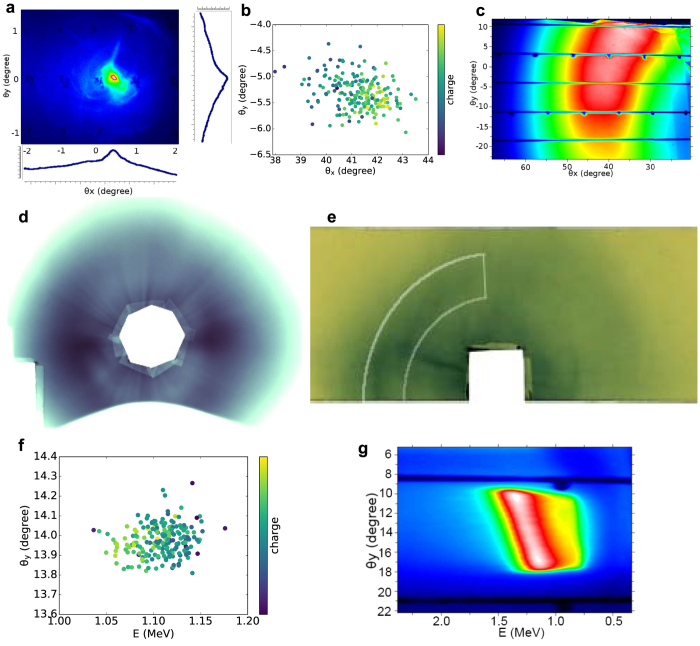
Electron beam angular and spectral distribution. (**a**) Single shot measurement on the forward LANEX. Mean emission angle (**b**) and accumulation (**c**) of 200 consecutive shots on the side LANEX.(**d**) Accumulation of 70 shots on a Gafchromic film placed 4 cm after the nozzle. (**e**) Single shot measurement on a bent Gafchromic film 4 cm from the nozzle. Energy measurement showing mean energy and angle (**f**) and accumulation (**g**) of 200 consecutive shots.

Experiments to investigate the properties and applications of wide-angle electrons have been conducted at the ALPHA-X beam line^18^ described in the Methods. Low-charge (~5 pC), low-divergence (~3 mrad) quasi-monoenergetic electron beams with 100–200 MeV mean energy are accelerated in the laser direction, with the simultaneous ejection of high-charge, relatively low-energy oblique and backward ejected beams. The experimental set-up is shown in Fig. 5, with further details presented in the Supplementary Section. The laser is linearly polarised in the horizontal direction (*x*). The spatial distribution of the forward electrons is measured on a LANEX screen placed 60 cm from the gas jet, observing high-energy bunches on a broad electron background halo (Fig. 6a). A second LANEX screen placed 7.5 cm from the gas jet at an angle of 55° from the laser axis is used to characterise oblique electrons, which are generated with high stability and reproducibility for a wide range of laser and plasma parameters.

The mean ejection angle for 200 consecutive shots is (41 ± 1)°, with rms horizontal divergence of (11.0 ± 0.5)° and 20% variation in charge (Fig. 6c). The dose distribution is measured by recording 100 shots on an 8.5 × 6 cm^2^ EBT2 Gafchromic dosimetry film^44^ wrapped in thin Al foil and placed 7.8 cm from the nozzle at a 38° angle from the laser axis. The maximum and average single-shot dose are 47 Gy and 27 Gy respectively. The corresponding charge is calculated using FLUKA^45^,^46^, a particle physics Monte Carlo simulation package, obtaining about 5 nC per shot. As these measurements only cover about a quarter of the beam, a 21 × 17.5 cm^2^ Gafchromic film is placed 4 cm in front of the nozzle with a 2 cm diameter hole to let the laser through. The distribution generated by 70 shots is presented in Fig. 6d, which is characterised by a ring at a 40° angle from the axis, with divergence of 12° and a higher charge density on the laser polarisation plane. The clipping at the bottom and left is caused by the gas nozzle mount. The total charge in the ring is estimated at 10 nC per shot. Figure 6e shows a single shot measurement obtained on Gafchromic film bent into a half-cylinder and placed in front of the nozzle. The cylinder radius is 4 cm, with its centre on the nozzle and axis coinciding with the direction of the gas flow. The maximum and average dose on the left half of the ring are 5.6 Gy and 3.2 Gy respectively, corresponding to a charge of 7.5 nC. These results are in excellent agreement with the numerical simulations of Figs 2 and 3, which predict an emission angle of approximately 40°, divergence of 10° and charge 4–10 nC for plasma densities in the range 1–2 × 10^19^ cm^−3^ and laser *a*_0_ 1–3.

The energy of oblique electrons is measured both with a magnetic spectrometer and using multi-layer filters, as described in Methods. The energy variation and sum of 200 consecutive shots on the spectrometer are shown in Fig. 6f and g, measuring beams on every shot with mean energy of (1.10 ± 0.25) MeV, energy spread of (0.5 ± 0.3) MeV and 34% variation in charge. The same statistics are obtained for 984 shots (see Supplementary Section), highlighting the high stability of side-electron beams and the excellent agreement with the theoretical results of Fig. 2. Figure 3 predicts somewhat larger energies, since it is obtained by integrating the particle distribution over a large angle, including the tail of high-energy electrons emitted closer to the axis. The energy dependence with angle is confirmed by the tilt on the high-energy side that is observed in Fig. 6g, which indicates a decrease in energy by about 0.5 MeV for an 8.6° angular change when moving away from the laser polarisation plane (*θ*_*y*_ = 0°), in agreement with Fig. 2. An exponential fit of measurements made on a LANEX screen behind a filter composed of 1 to 5 layers of 250 μm Al sheet provides an energy of 1.1 MeV, in agreement with the spectrometer results.

Oblique electrons are produced for plasma densities between 1 and 2 × 10^19^ cm^−3^ by changing the nozzle backing pressure and height of laser focus above the nozzle, which show no significant difference in the angular and spectral distributions. When the laser energy is reduced, we only observe a decrease in charge. These results are in agreement with the theoretical predictions and show that oblique electrons are relatively insensitive to laser and density fluctuations, making this acceleration mechanism useful for applications requiring stable, high-charge, low-energy electron beams. An example of an imaging measurement is presented in the Supplementary Section (Figure S.13).

**Figure 7:**
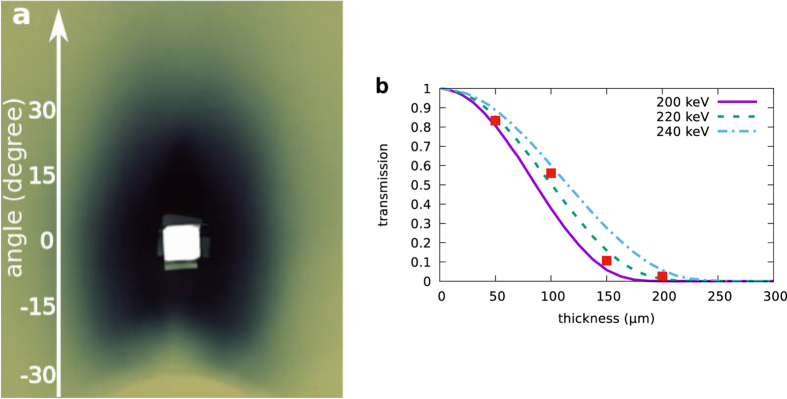
Backward-accelerated electrons characterisation using Gafchromic films. (**a**) Spatial profile and (**b**) energy measurement using a filter with 1 to 4 layers of 50 μm Al sheets (square symbols); curves show the Al transmission for different electron energies obtained with Geant4.

In addition to forward and oblique beams, LWFAs also eject electrons counter to the laser propagation direction. The spatial distribution produced by 60 shots on a Gafchromic film placed 5 cm upstream of the nozzle is shown in Fig. 7a, observing a slightly elliptical beam with the short and long axis subtending respectively a 30° and 40° half-angles, with charge of 3.5 nC per shot. The beam is partially obscured in the lower half of the image because the nozzle casts a “shadow”, and a 1cm × 1 cm hole is cut in the film to let the laser through. The mean energy is 200 keV, which is obtained recording 10 shots on a Gafchromic film placed behind 1 to 4 layers of 50 μm Al foils positioned above the beam axis (Fig. 7b). The total energy is of the order of 100 s μJ. Additional measurements have been performed, recording between 1 and 100 shots, which give similar values for the average charge per shot and also stable emission.

## Discussion

Numerical simulations and experiments show that LWFAs emit high-charge, low-energy side-electron beams that are insensitive to the initial conditions over a large range of laser and plasma parameters. These wide-angle beams are stable and suitable for applications that require high fluxes of electrons or x-rays. On the other hand, oblique electrons carry a large fraction of laser energy out of the plasma, limiting the efficiency of LWFAs for the production of high-energy forward electron beams. This is a particular concern for LWFAs based on capillaries because of potential material damage to the capillaries and the production of significant bremsstrahlung radiation. Oblique beams can also be a source of unwanted radiation if not properly dumped. Knowledge of their properties is therefore also important for the study and application of high-energy forward beams since their trapping processes are closely associated to the side-electron emission.

In conclusion, LWFAs can simultaneously produce three electron beams: ultra-short electron bunches with MeV-GeV energies and pC-level charge, co-propagating with the driving laser; beams with MeV energy and nC-level charge emitted in a broad forward directed cone; and beams with 100 s of keV energy and nC-level charge accelerated backwards. Here, we have reported the first full characterisation of oblique and backward electrons, showing that stable beams with 1–2 MeV energy and total charge in excess of 10 nC are ejected in a cone with 30°–60° angle from the laser propagation axis. Simulations show that oblique electrons are accelerated as they stream around the cavity (ion “bubble”) void of electrons, which trails the intense laser pulse as it interacts with the underdense plasma. We also report the first characterisation of backward electrons, which are mostly ejected from the plasma entrance, where an energy of 200 keV and 3.5 nC charge are measured. Unlike forward ejected electron beams, which require careful tuning of laser and plasma parameters for stable operation, oblique and counter-propagating electrons are generated with high reproducibility and stability, with little dependence on laser and plasma parameters, making these beams suitable for applications like dosimetry, radiolysis or radiobiology where high-charge low-energy electron beams are required. Our analysis indicates that existing theoretical models of the plasma bubble formations are insufficient to describe the observed side and backward electron emission, i.e. the dynamic bubble evolution needs to be included. Knowledge of the existence and properties of these beams is also important in experiments using high-energy forward electron beams, for example to reduce unwanted bremsstrahlung radiation or to prevent incorrect charge measurements if the broad halo emitted in the forward direction is not removed using filters or magnets. This information is pertinent to the design of staged high energy accelerators, where knowledge of the overall efficiency is required.

## Methods

### PIC simulations

The interaction of a high-power laser with a helium gas jet is simulated both in 3D and 2D geometry using the particle-in-cell (PIC) code OSIRIS^47^. The laser propagates along the *x*_1_ direction, with a longitudinal profile described by a sin^2^ function with a full width at half maximum varying between 20 and 30 fs. The transverse profile is Gaussian and the beam size *w*_0_ (radius at the 1/*e*^2^ intensity point) is varied between 5 and 10 μm. The focal plane is located at the entrance of a pre-ionised plasma with isotropic thermal energy of 20 eV and constant density profile with a linear up-ramp of length 40 μm in 3D and 60 μm in 2D. The laser is linearly polarised in the *x*_2_ (horizontal) direction for 3D simulations and in the *x*_3_ direction for 2D simulations. Simulations are performed in a box moving at the speed of light. The grid size in 3D is 50 μm × 40 μm × 40 μm with 1560 × 160 × 160 cells, corresponding to a resolution of *λ*_*L*_/25 in the longitudinal (*x*_1_) direction and *λ*_*L*_/3.2 in the transverse direction, respectively. In 2D several configurations are employed to ensure that the grid size and resolution have no effect on the properties of side electrons. Unless otherwise specified, the results presented here are obtained for a 60 μm× 80 μm moving window with 3450 × 2400 cells, corresponding to a resolution of *λ*_*L*_/46 in the longitudinal (*x*_1_) direction and *λ*_*L*_/24 in the transverse direction. In the calculation of the properties of oblique electrons, high-energy forward electrons and low-energy plasma electrons are filtered out from all simulations by selecting only electrons with longitudinal momentum 1.4 < *p*_1_/*mc* < 50 and transverse position *r* > 5 μm. Further details and movies of Figs 1 and 4 are given in the Supplementary Section.

### Laser-wakefield accelerator

Experiments to investigate the properties and possible applications of wide-angle electrons have been conducted at the Advanced Laser-Plasma High-energy Accelerators towards X-rays (ALPHA-X) beam line. A Ti:sapphire Chirp Pulse Amplification (CPA) laser system delivers 35 fs, 800 nm pulses with on-target energy of 900 mJ. The laser beam is focused onto a gas jet by an *f*/18 spherical mirror to a vacuum spot size of 20 μm (radius at the 1/*e*^2^ intensity point). A supersonic He gas jet is produced by a 2 mm diameter nozzle with plasma density 1–2 × 10^19^ cm^−3^ (ref. 48). The laser peak intensity is 2 × 10^18^ W/cm^2^, corresponding to a normalised vector potential *a*_0_~1, which grows to *a*_0_ > 3 due to relativistic self-focusing of the beam causing self-compression to a ~5 μm radius channel. The laser is polarised in the horizontal plane (*x*). Additional details and measurements are presented in the Supplementary Section.

### Electron energy diagnostics

The energy of side-electrons is measured using two diagnostic methods: a magnetic spectrometer and multi-layer filters. The spectrometer has an operating range of approximately 0.5–3 MeV and is composed of two dipole magnets separated by a 1 cm gap producing a 200 mT magnetic field. A 2 mm wide copper slit is placed in front of the magnets and a LANEX screen wrapped in thin Al foil is on the back. The spectrometer is placed 5 cm from the nozzle and oriented at an angle of 45 to the laser axis. The electron energy is also measured using a filter composed of 1 to 5 layers of 250 μm Al sheets placed 6.4 cm from the nozzle at an angle of 38° to the laser propagation axis (the same position of the EBT2 film in Fig. 5). A LANEX screen is used for detection. The energy is determined using a calibration curve obtained by modelling the system with Geant4^47^. A multi-layer filter is also used to measure the energy of backward electron. Because of the lower energy, the Al sheet thickness is reduced to 50 μm, employing 1 to 4 layers. A Gafchromic film is used for detection. Sample images are presented in Supplementary Figure 13.

### Data availability

Data associated with research published in this paper is available at http://dx.doi.org/10.15129/ec551569-89e6-4f48-bcc0-bd3bae0455a1.

## Additional Information

**How to cite this article**: Yang, X. *et al*. Three electron beams from a laser-plasma wakefield accelerator and the energy apportioning question. *Sci. Rep.* **7**, 43910; doi: 10.1038/srep43910 (2017).

**Publisher's note:** Springer Nature remains neutral with regard to jurisdictional claims in published maps and institutional affiliations.

## Supplementary Material

Supplementary video 1

Supplementary video 2

Supplementary Information
