# Supplementary material for: Three electron beams from a laser-plasma wakefield accelerator and the energy apportioning question
Source: Sci Rep. 2017 Mar 10;7:43910. doi: 10.1038/srep43910 (PMC5345066; doi:10.1038/srep43910)
Supplement: Supplementary Information [file srep43910-s3.pdf]

Three electron beams from a laser-plasma wakefield accelerator and the energy apportioning question

X. Yang[1,†], E. Brunetti[1], D. Reboredo Gil[1], G.H. Welsh[1], F.Y. Li[1], S. Cipiccia[1], B. Ersfeld[1], D.W. Grant[1], P.A. Grant[1], M.R. Islam[1], M.P. Tooley[1], G. Vieux[1,2], S.M. Wiggins[1], Z.M. Sheng[1,3], and D.A. Jaroszynski[1,*]

[1]SUPA, Department of Physics, University of Strathclyde, Glasgow, G4 0NG, UK
[2]Institute of Physics of the ASCR, ELI-Beamlines, Na Slovance 2, 182 21 Prague, Czech Republic
[3]Laboratory of Laser Plasmas and Department of Physics and Astronomy, Shanghai Jiao Tong University, Shanghai 200240, China
[†]Present address: Department of Physics, Capital Normal University, Beijing 100048, China
[*]Correspondence and requests for materials should be addressed to E.B. (email: enrico.brunetti@strath.ac.uk) or D.A.J. (email: d.a.jaroszynski@strath.ac.uk)

Numerical simulations

The interaction of a high-power laser with a helium gas jet is simulated both in 3D and 2D geometry with the particle-in-cell (PIC) code OSIRIS[1]. The laser propagates along the x_1 direction, with a longitudinal profile taken as a $\sin^2$ function with full width at half maximum in the range between 20 and 30 fs. The transverse profile is Gaussian and the beam radius w_0 is varied between 5 and 10 µm. The focal plane is located at the entrance of a pre-ionized plasma with constant density profile and linear up-ramp of length 40 µm in 3D and 60 µm in 2D. The laser is linearly polarised in the horizontal (x_2) direction for 3D simulations and in the x_3 direction for 2D simulations. The intensity I_0 is expressed in terms of the normalised vector potential $a_0 = 8.5\times10^{-10}\lambda_L[\mu\text{m}](I_0[\text{W/cm}^2])^{1/2}$, with λ_L the laser wavelength. Here $\lambda_L = 0.8\,\mu$m. Simulations are performed in a box moving at the speed of light. In 3D the grid size is $50\,\mu\text{m}\times40\,\mu\text{m}\times40\,\mu\text{m}$ with $1560\times160\times160$ cells, corresponding to a resolution of $\lambda_L/25$ in the longitudinal (x_1) direction and $\lambda_L/3.2$ in the transverse direction. In 2D several configurations are employed to study the effect of grid size and resolution on the properties of side electrons. Energy and angle are found to change by only a few percent when going from a resolution of $(\lambda_L/25, \lambda_L/3.2)$ to $(\lambda_L/72, \lambda_L/24)$. Unless otherwise specified, results presented here are obtained for a $60\,\mu\text{m}\times80\,\mu\text{m}$ moving window with 3450×2400 cells, corresponding to a resolution of $\lambda_L/46$ in the longitudinal (x_1) direction and $\lambda_L/24$ in the transverse direction. When the properties of wide-angle electrons are calculated, high-energy forward electrons and low-energy plasma electrons are filtered out from all simulations by selecting only electrons with longitudinal momentum $1.4 < p_1/mc < 50$ and transverse position $r > 5\,\mu$m. Comparison with 2D simulations performed in a fixed $1030\,\mu\text{m}\times300\,\mu\text{m}$ box shows that this choice of filtering and moving window offers a good compromise between accuracy and computational time.

Although the non-linear evolution of laser and plasma cannot be fully reproduced in 2D geometry[2], 2D simulations allow quick scans of a large number of parameters and offer some guidance on the qualitative behaviour of the system. This is because the power contained in a pulse slice with transverse size w is proportional to $a_0^2w^2$ in 3D, and a_0^2w in 2D[3]. The reduced power in 2D geometry results in underestimating the effect of laser self-focusing, which is a key process governing system evolution[4]. To compensate for this power discrepancy, 2D simulations are performed with a scaled a_0. It will be shown that with this correction 2D and 3D simulations predict similar energies and ejection angles for oblique electrons, in addition to similar maximum energies for the forward accelerated electron beams. Fundamental differences, however, still remain, especially for high plasma densities and laser intensities, when bubble size and shape changes significantly during propagation. For example, 2D simulations often predict a large growth in bubble size, whereas in 3D the accelerating structure becomes asymmetric and quickly collapses.

The properties of oblique electrons obtained from 3D and 2D simulations after 0.5 mm interaction are presented in Figure S.1 for a pre-ionised plasma with density $2\times10^{19}\,\text{cm}^{-3}$ (left and middle column) and for He gas with neutral density $1\times10^{19}\,\text{cm}^{-3}$ (right column). Plots in the left column have been obtained for laser $a_0 = 2$ and spot size $w_0 = 10\,\mu$m and show a typical distribution with electrons clustering along the laser polarisation plane. A similar pattern is observed in the middle column, which is obtained for laser $a_0 = 3$ and spot size $w_0 = 7\,\mu$m, corresponding to Figure 2d in the main paper. The clustering along the polarisation plane is stronger in neutral gas, as shown by

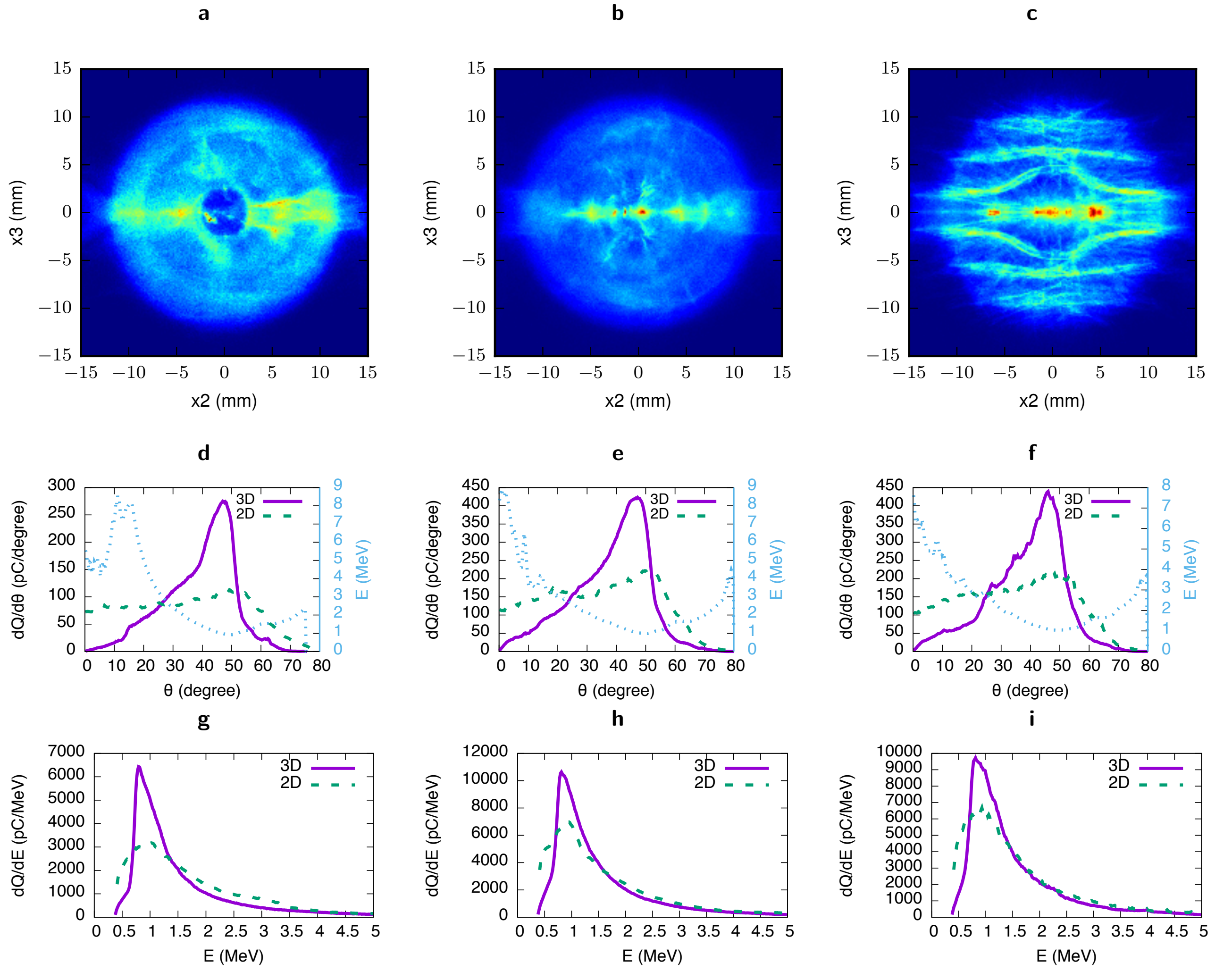

Figure S.1. Simulated spatial (a-c), angular (d-f) and spectral (g-i) distribution of oblique electrons projected on a screen located 10 mm downstream from the plasma. Plots in the left and middle columns are for pre-ionised plasma with density $2 \times 10^{19}\,\mathrm{cm}^{-3}$, whereas plots on the right column are for He gas with neutral density $1 \times 10^{19}\,\mathrm{cm}^{-3}$. The laser parameters are $a_0 = 2$, $w_0 = 10\,\mu\mathrm{m}$ (left) and $a_0 = 3$, $w_0 = 7\,\mu\mathrm{m}$ (middle and right). Curves in 2D simulations are normalized to the total charge obtained in 3D. Dotted lines in figures (d-f) show the energy dependence on angle from 3D simulations.

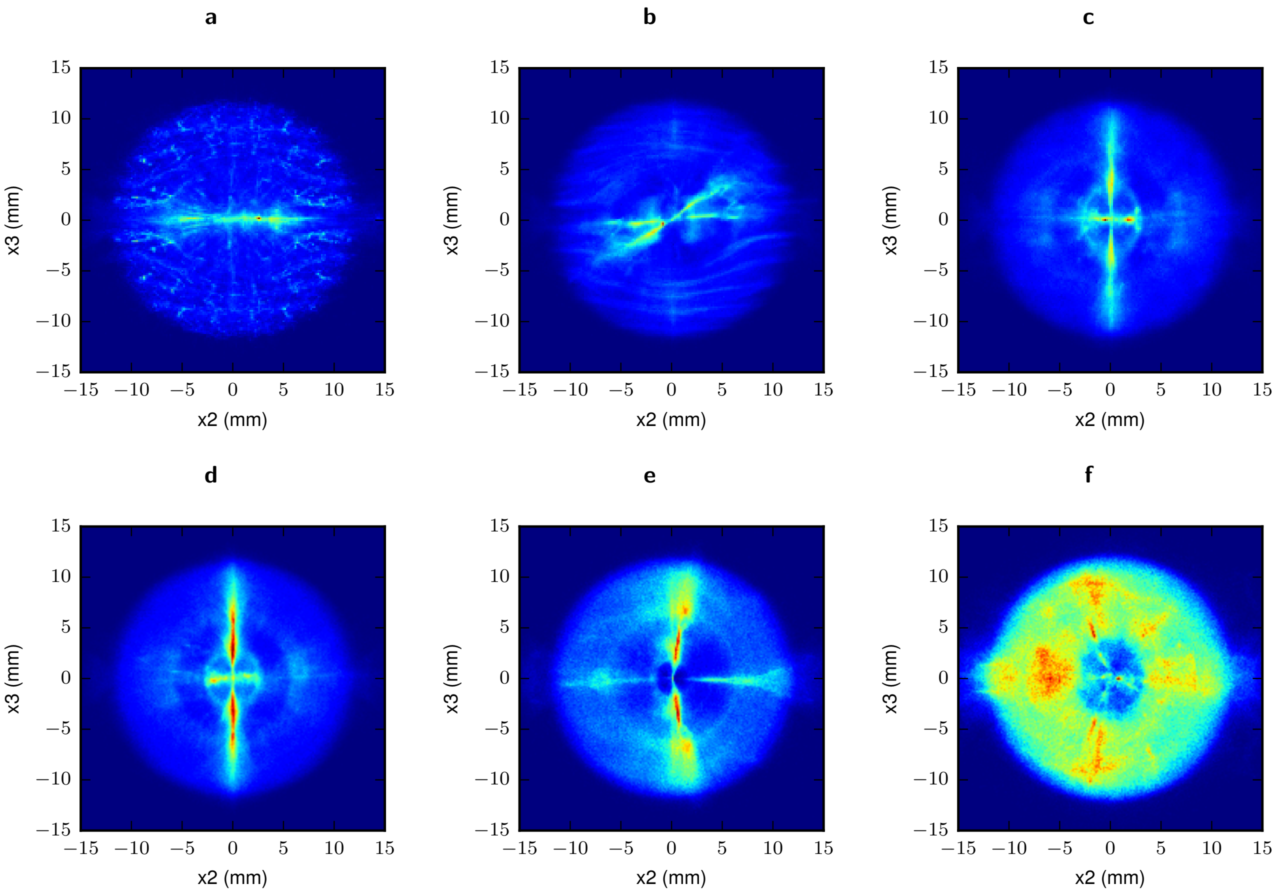

Figure S.2. Spatial distribution of oblique electrons projected on a screen located 10 mm downstream from the plasma for a pre-ionised plasma with density 2×10^{19} cm^{-3} and laser spot size $w_0 = 7$ µm. a) Initial plasma temperature of 0 eV and laser $a_0 = 2$. b) Initial plasma temperature of 0 eV along x_3 and 20 eV along x_1, x_2 and laser $a_0 = 2$ c) Initial plasma temperature of 5 eV and laser $a_0 = 2$. Initial plasma temperature of 20 eV and laser d) $a_0 = 2$, e) $a_0 = 2.2$ and f) $a_0 = 2.5$.

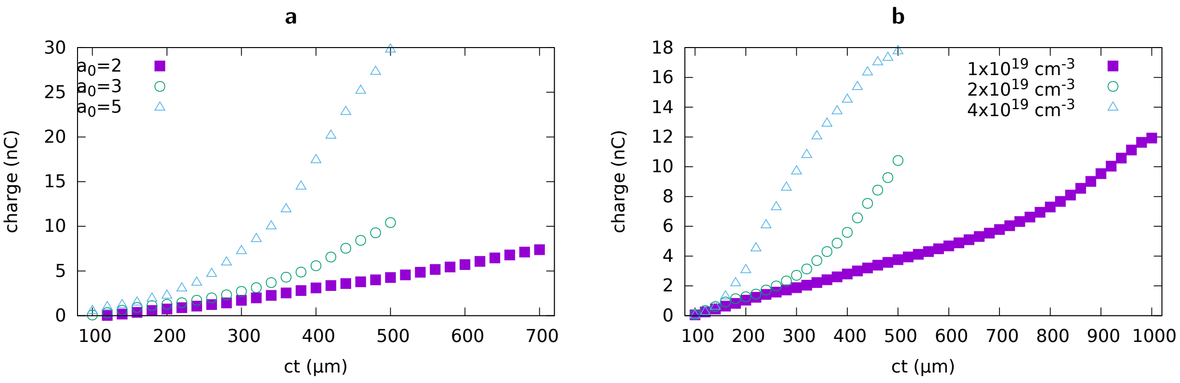

Figure S.3. Charge of oblique electron beams versus propagation distance ct for laser $w_0 = 7$ µm and a) varying a_0 at density 2×10^{19} cm^{-3} and b) varying density at $a_0 = 3$.

the plots in the right column, obtained for the same laser parameters $a_0 = 3$ and $w_0 = 7\,\mu\text{m}$. In Osiris, electrons released through ionisation have zero temperature, although the ADK model predicts a momentum distribution with Gaussian shape and widths of a few eVs transversely to the laser polarisation direction[5,6]. Experimental and theoretical studies suggest that electrons are born with a non-zero momentum spread also in the direction parallel to the laser electric field[6,7]. Further heating in the laser field can increase the temperature to tens of eV[8]. Similar temperatures are also found in pre-formed plasmas. For example, hydrogen-filled capillary waveguides have plasma temperature around 5 eV[9]. Figure S.2 shows that the clustering pattern is strongly affected by the initial electron momentum distribution, especially in the vertical direction for a horizontally polarised laser beam. Clustering is preferentially along the laser polarisation direction for electrons initially at rest (Figure S.2a) or with thermal spread only in the x_1 and x_2 directions (Figure S.2b). On the other hand, for an isotropic thermal spread, patterns can develop across the transverse plane, with little dependence on the temperature, as shown for 5 eV (Figure S.2c) and 20 eV (Figure S.2d). The variation with laser parameters is stronger (Figure S.2e and S.2f). Clustering patterns also depend on the carrier-envelope phase of the laser pulse. For example, Figure S.2b shows a feature running from the bottom-left to the top-right with respect to the x_3 axis. If the carrier envelope phase is shifted by 90°, this feature is flipped about the x_2 axis and runs from the bottom-right to the top-left. A carrier envelope phase shift by 180° restores it to the original orientation.

The emission rate is presented in Figure S.3, which shows that high charge is achievable over short propagation distances, especially for large laser a_0 and high plasma densities.

Semi-analytical model

The electromagnetic fields produced by a ionic sphere moving at constant velocity $\mathbf{v_b} = v_b\hat{\mathbf{x}}$ in a plasma with electron density n_e can be derived analytically in the quasi-static approximation to obtain[10]

$$
\begin{aligned}
E_x &= \frac{m_e\omega_p^2}{e}\frac{x - v_b t}{2}, & B_x &= 0, \\
E_y &= \frac{m_e\omega_p^2}{e}\frac{y}{4}, & B_y &= \frac{m_e\omega_p^2}{ec}\frac{z}{4}, \\
E_z &= \frac{m_e\omega_p^2}{e}\frac{z}{4}, & B_z &= -\frac{m_e\omega_p^2}{ec}\frac{y}{4},
\end{aligned} \tag{1}
$$

with $\omega_p = \sqrt{n_e e^2/(m_e\epsilon_0)}$ the plasma frequency, e the magnitude of the electron charge, m_e the electron mass, ϵ_0 the vacuum permittivity and c the speed of light in vacuum. Terms of order $1/\gamma_b^2$, with $\gamma_b = 1/\sqrt{1-\mathbf{v}_b^2/c^2}$ the relativistic factor, are neglected. It is assumed that the cavity is surrounded by a thin electron sheath of width d, which is modelled by multiplying the electromagnetic fields by the shape function[10]

$$
S(r) = -\frac{1}{2}\left[\tanh\left(\frac{r - r_b}{d}\right) - 1\right],
$$

with $r = \sqrt{(x - v_b t)^2 + y^2 + z^2}$ and r_b the cavity radius. Here a fixed value $d = 0.5\,\mu\text{m}$ is used. The force acting on electrons is $F = -eS(r)(\mathbf{E} + \mathbf{v}\times\mathbf{B})$ and trajectories are obtained by numerically solving the relativistic equations of motion.

Energy and angle

The dynamics of electrons initially at rest at $x = 15\,\mu\text{m}$, $z = 0$ and interacting with a single non-evolving bubble is investigated for different bubble parameters, which are independently varied, although, as it will be shown below, this is not always possible in practice.

Sample trajectories of electrons initially displaced by $y = 3$ to $7\,\mu\text{m}$ are shown in Figure S.4a and S.4b for bubble radius $r_b = 6\,\mu\text{m}$ and plasma density $n_e = 2\times10^{19}\,\text{cm}^{-3}$, with the final energy and angle distributions for different bubble sizes and fixed $v_b = 0.97778c$ ($\gamma_b = 4.8$) presented in Figure S.4c and Figure S.4d. The final energy and angle depend on the initial transverse displacement from the axis, with electrons located approximately at the cavity radius accelerated to higher energies and ejected at smaller angles, up to a maximum energy of about 20 MeV, capped by the onset of injection.

The dependence of the electron beam mean energy and angle on the bubble radius is presented in Figure S.5a and S.5b for different bubble speeds and a fixed accelerating gradient, which corresponds to a plasma density $n_e = 2\times10^{19}\,\text{cm}^{-3}$. Higher energies are achievable for larger bubble sizes and higher bubble speeds, when the

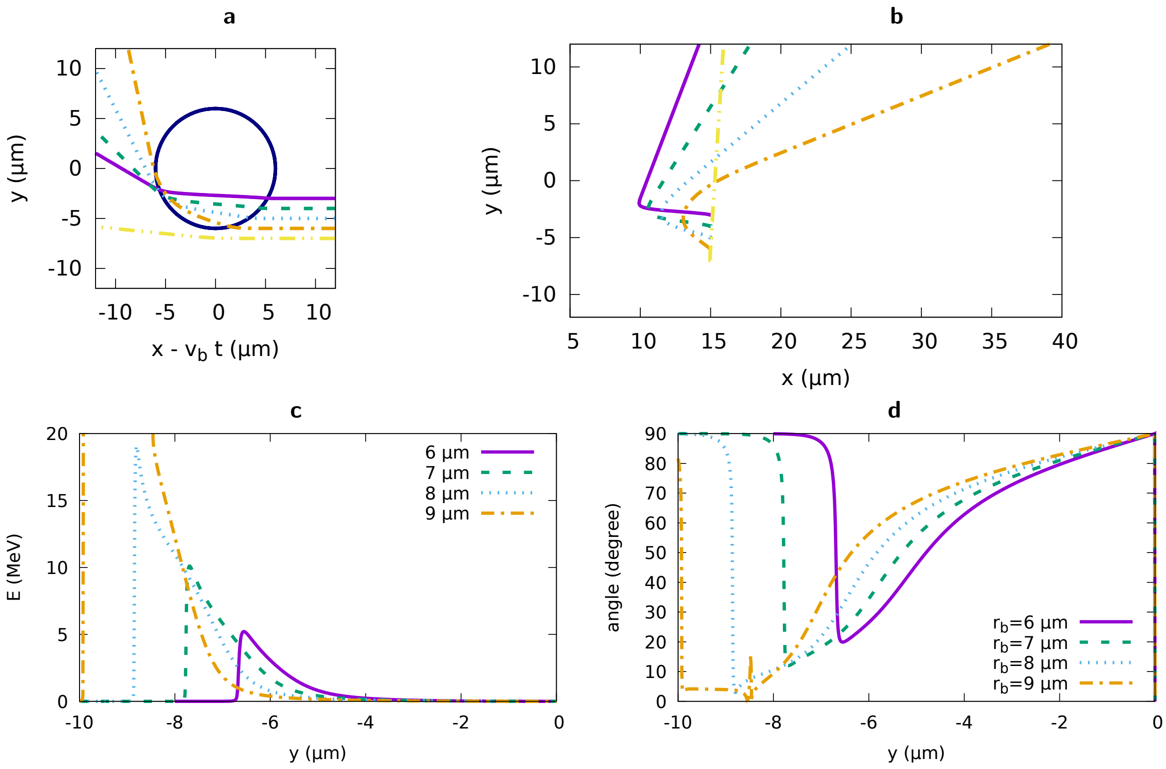

Figure S.4. Electron evolution using Kostyukov's model for a single spherical bubble and plasma density $n_e = 2 \times 10^{19}\,\mathrm{cm}^{-3}$. Sample trajectories plotted in a Galileian frame moving at the bubble speed v_b (a) and in the lab frame (b) for $r_b = 6\,\mu\mathrm{m}$ and $v_b = 0.97778c$ ($\gamma_b = 4.8$). Final energy (c) and angle (d) versus initial displacement for $v_b = 0.97778c$ and bubble radius between 6 and 9 µm.

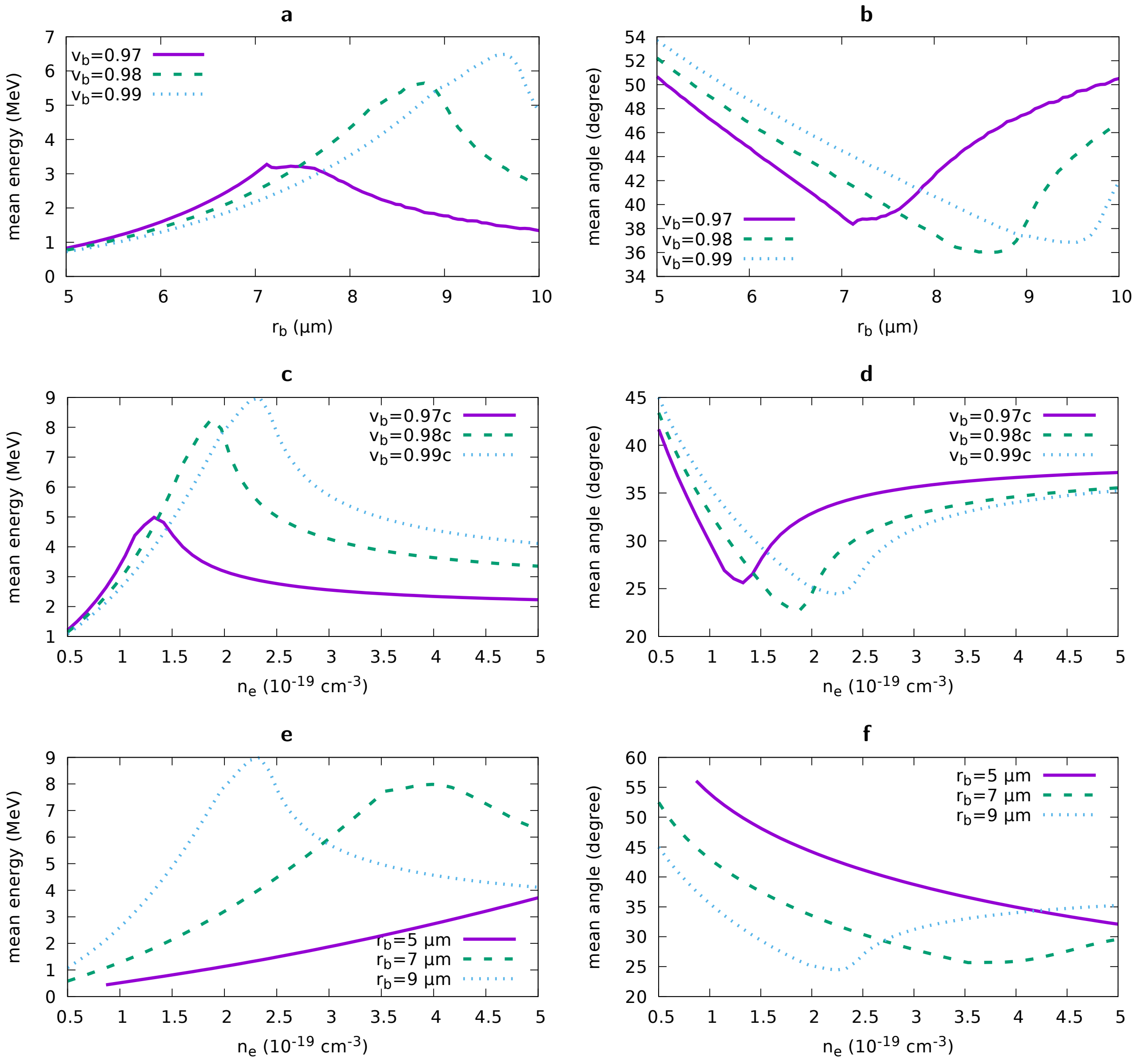

Figure S.5. Electron evolution using Kostyukov's model for a single spherical bubble. Mean energy (a) and angle (b) versus bubble radius for plasma density $n_e = 2 \times 10^{19}\,\mathrm{cm}^{-3}$ and bubble speeds of $0.97c$, $0.98c$ and $0.99c$. Mean energy (c) and angle (d) versus plasma density for $r_b = 9\,\mu\mathrm{m}$ and bubble speeds of $0.97c$, $0.98c$ and $0.99c$. Mean energy (e) and angle (f) versus plasma density for bubble speed of $0.99c$ and $r_b = 5$, 7 and $9\,\mu\mathrm{m}$. Mean values are calculated for electrons with energy between 0.1 and 20 MeV.

acceleration lengths are longer, but the difference is small, due to the onset of injection, which removes high energy particles. The accelerating gradient is proportional to the density (equation 1), therefore the beam energy increases with density for fixed bubble radius and speed, but the growth is capped by injection, as shown in Figure S.5c and S.5d for $r_b = 9\,\mu m$. For different bubble sizes the threshold density for injection changes, but with little effect on the range of attainable energies (Figure S.5e).

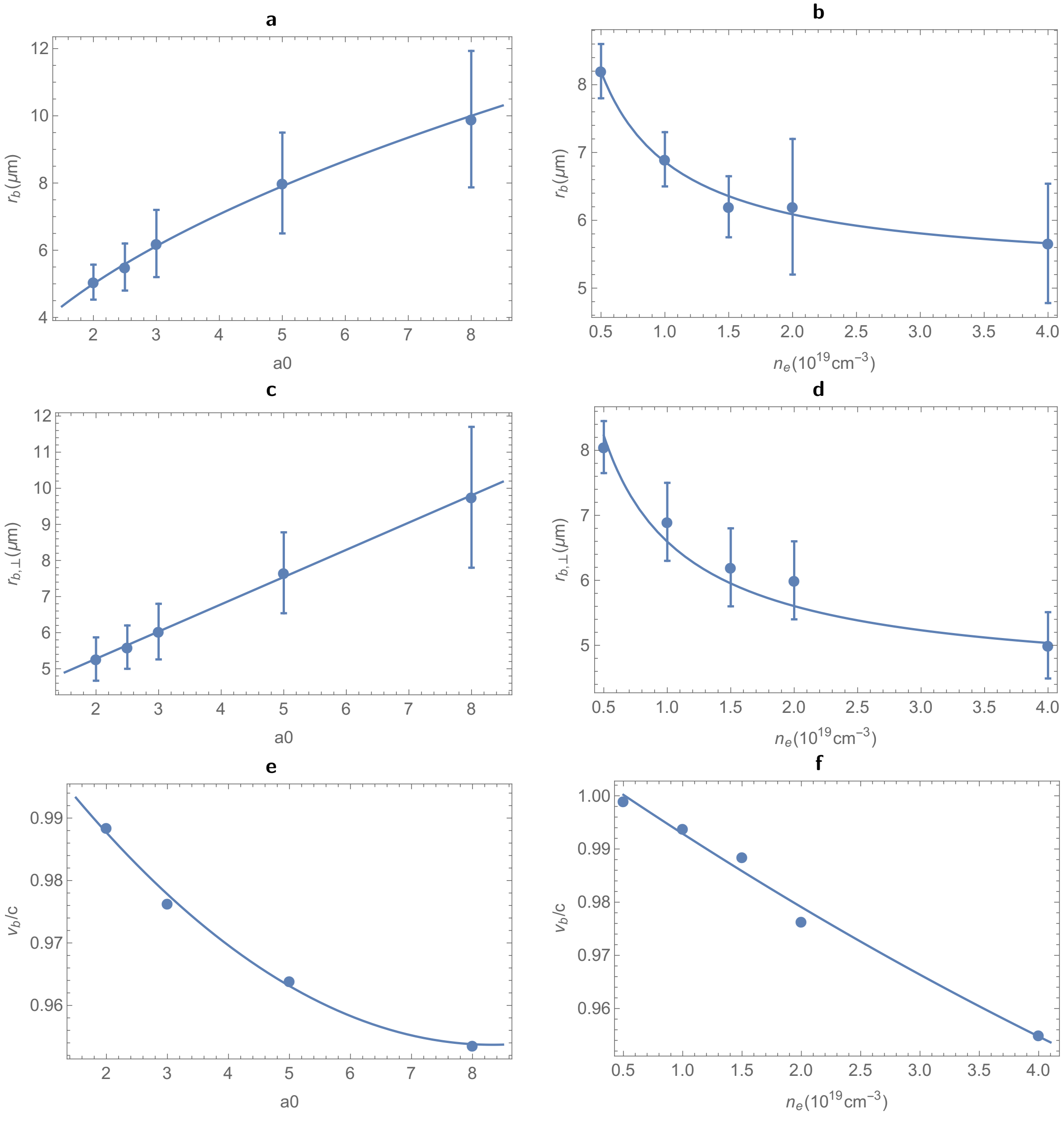

Figure S.6. Dependence on laser a_0 and plasma density n_e of average bubble radius (a-b), transverse radius (c-d) and bubble speed (e-f) as obtained from 3D PIC simulation for laser spot size $w_0 = 7\,\mu m$. Figures (a,c,e) are for $n_e = 2\times10^{19}\,cm^{-3}$ and figures (b,d,f) for $a_0 = 3$.

The bubble size and speed are determined by the laser and plasma parameters. Their dependence on laser a_0 and plasma density obtained from 3D PIC simulations is presented in Figure S.6 for laser beam waist $w_0 = 7\,\mu m$. Since the bubble shape changes during propagation, quantities shown here are averages over the first 0.5 mm propagation, with the error bars representing the standard deviation. The bubble radius r_b, calculated as the average of the longitudinal and transverse radii, scales as $r_b = r_{0a}\sqrt{a_0}$ (Figure S.6a) and $r_b = r_0\sqrt{1+n_{e,0}/n_e}$ (Figure S.6b), with r_{0a}, r_0 and $n_{e,0}$ parameters determined by fits. The transverse radius evolution is similar, but the scaling with a_0 is

closer to a linear growth (Figure S.6c). The speed of the back of the bubble is shown in Figure S.6e and S.6f, with the curves representing quadratic fits. In first approximation, the bubble speed is given by the laser group velocity in plasma $v_g = c\sqrt{1-\omega_p^2/\omega^2}$, but for high intensities the non-linear evolution of the laser beam causes it to slow down[11].

Figure S.5a indicates that side-electron mean energies beyond 5 MeV should be achievable for high bubble speeds. However, Figure S.6f shows that these conditions are not realisable in practice, since high speeds require low density plasmas, where the low accelerating gradient limits the energy to the 1–2 MeV range (Figure S.5e). A small energy boost can be obtained by increasing the laser spot size, and therefore the bubble radius, but stable propagation would require increasing a_0 to satisfy the condition $r_b \approx w_0 \approx 2\sqrt{a_0}\,c/\omega_p$, which minimises bubble size oscillations[11]. As discussed above, increasing a_0 reduces the bubble speed and therefore the beam energy. The narrow range of achievable energies makes side-electron beams relatively insensitive to laser and plasma parameters, as observed in the PIC simulations presented here and in Figure 2 and 3 of the paper.

Charge

Here we describe a rudimentary model to estimate the side-electron beam charge using the results of the previous section. As shown in Figure S.4, obliquely ejected electrons originate from a cylindrically symmetric region located approximately a bubble radius off-axis. The beam charge is estimated to be $Q = q_e n_e V$, with q_e the electron charge, n_e the plasma density and V the volume of the hollow cylinder, which has a thickness determined by the laser and plasma parameters. However, it should be noted that this method has many limitations, such as an incomplete modelling of the field and bubble evolution, which also leads to inaccurate predictions of the injection threshold. Furthermore, the laser field is not included, even though it can push electrons off-axis to positions where the energy gain is larger. This model also does not account for beam-loading by accelerated beams in the bubble, which will also affect the flow of current around the bubble. Nevertheless, the dependence of the side-electron beam charge on laser a_0 and plasma density obtained from 3D PIC simulations (Figure 3e and 3f of the main paper) can be reproduced.

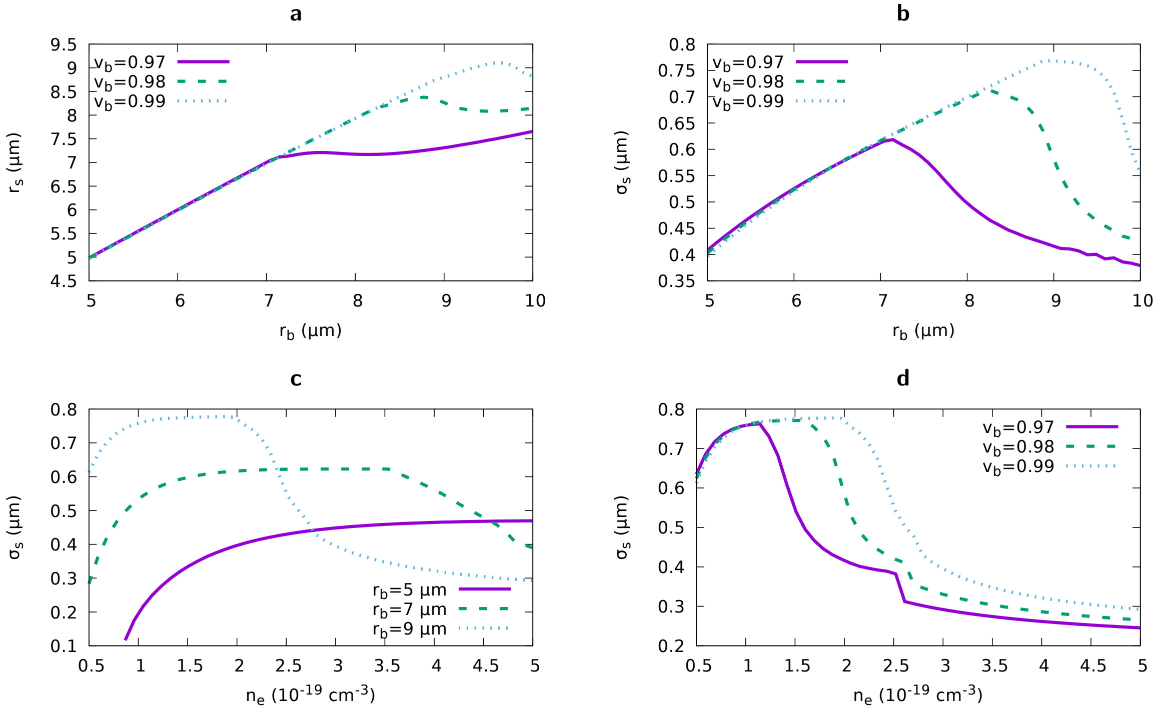

Figure S.7. a) Mean position and b) rms size of the side-electron source region as a function of the bubble radius for different bubble speeds and fixed accelerating gradient, corresponding to $n_e = 2\times10^{19}\,\mathrm{cm}^{-3}$. Source rms size versus density for c) different bubble radius and fixed bubble speed $v_b = 0.99c$ and d) different bubble speeds and fixed bubble radius $r_b = 9\,\mu\mathrm{m}$. Only electrons with energy between 0.4 and 20 MeV are considered.

The scaling of the beam charge with a_0 is estimated by assuming that the cylinder outer radius r_o is given by the transverse bubble radius plotted in Figure S.6c, which is proportional to a_0 for the parameter range considered

here. The cylinder inner radius r_i is also assumed to grow linearly with a_0, although at a slower rate, to match the increase in source size observed in Figure S.7b. With these assumptions, the base of the hollow cylinder has area $\pi(r_o^2 - r_i^2)$ and grows quadratically with a_0. For a constant height and density, the cylinder volume, and therefore the beam charge, also grows quadratically with a_0, recovering the results of Figure 3e in the main paper, which is reproduced in Figure S.8a together with a curve obtained for a cylinder with thickness growing from 0.2 µm for $a_0 = 3$ to 0.9 µm for $a_0 = 8$ and with 0.4 mm height, matching the emission length in Figure S.3 over the first 0.5 mm propagation distance.

The scaling of the beam charge with the plasma density n_e is estimated by assuming that the cylinder outer radius r_o is given by the transverse bubble radius $r_b = r_0\sqrt{1+n_{e,0}/n_e}$ plotted in Figure S.6d. According to Figure S.7b, the source width should decrease with increase in density because of the smaller bubble radius. However, Figure S.7c and S.7d indicates that this effect is balanced by the larger accelerating gradient, which causes the width to remain approximately constant over a large range, with a drop only at low densities. Therefore, the volume of ejected electrons changes little and the beam charge is proportional to the density, recovering the results of Figure 3f in the main paper, which are also reproduced in Figure S.8b together with a curve obtained for a cylinder with thickness increasing from 0.1 µm at 5×10^{18} cm^{-3} to 0.25 µm at 4×10^{19} cm^{-3} and with 0.4 mm height.

Experimental results

Experiments to investigate the properties and possible applications of wide-angle electrons have been conducted at the Advanced Laser-Plasma High-energy Accelerators towards X-rays (ALPHA-X) beam line[12]. A Chirp Pulse Amplification (CPA), Ti:sapphire laser system delivers 35 fs, 800 nm pulses with on-target energy of 900 mJ. The laser beam is focused on a gas jet by an $f/18$ spherical mirror to a vacuum spot size of 20 µm (radius) at the $1/e^2$ intensity point. A supersonic He gas jet is produced by a 2 mm diameter nozzle with plasma density 1–3×10^{19} cm^{-3}. The laser peak intensity is 2×10^{18} W/cm^2, corresponding to a normalised vector potential $a_0 \sim 1$, which grows to $a_0 > 3$ due to relativistic self-focusing of the beam causing self-compression to a ∼5 µm radius channel. The laser polarisation direction is horizontal (x). Low charge (∼5 pC), low divergence (∼3 mrad) quasi-monoenergetic electron beams with 100-200 MeV mean energy are produced along the laser propagation direction, simultaneously with the emission of high-charge, low-energy oblique electron beams. As shown in Figure 5 (main paper), a scintillating LANEX screen wrapped in Al foil and placed 60 cm from the gas jet along the laser propagation direction is used to measure the spatial distribution of forward electrons. A second LANEX screen placed at a distance of 7.5 cm from the gas jet at a 55° angle with respect to the laser propagation axis is used to characterise oblique electrons. These off axis electrons accelerated outside the bubble could easily be mistaken for electrons accelerated inside the bubble.

The angular variation of the oblique electron beam for 200 consecutive shots is presented in Figure S.9, registering a mean ejection angle of $(41\pm1)°$, with rms horizontal divergence of $(11.0\pm0.5)°$ and 20% variation in charge. Sample energy spectra of the side electrons measured with the magnetic spectrometer are presented in Figure S.10. The energy variation for 5 runs containing a total of 984 shots is presented in Figure S.11, registering a mean energy of (1.10 ± 0.25) MeV, with energy spread of (0.52 ± 0.03) MeV and 34% variation in charge.

Measurements made on a LANEX screen behind a multi-layer Al filter (Figure S.12a) show that the thickest layer (1.25 mm at the top) blocks most of the side-electrons, whereas 90% of the beam is transmitted by the thinnest layer (0.25 mm at the bottom). An exponential fit for 5 layers provides an energy of 1.1 MeV. The mean energy of backward electron is obtained from an exponential fit of 10 shots recorded on a Gafchromic film placed behind 1 to 4 layers of 50 µm Al foils positioned above the beam axis (Figure S.12b). An exponential fit provides an energy of 200 keV.

To demonstrate a possible application of off-axis beams from LWFAs, we have utilised the wide-angle electrons to image a 6×4.5 cm^2 hard drive controller board with 1 mm thick base (Figure S.13a), placed at a distance of 5 cm from the nozzle and orthogonal to the side electron beam. Figure S.13b is obtained for 20 shots on a Gafchromic film and Figure S.13c is obtained for a single shot on an imaging plate (Fuji BAS-SR), which has higher sensitivity.

Video Legends

Side-electron video

Video corresponding to Fig. 1 (main paper), showing results of a 2D OSIRIS simulation of the emission of low-energy electrons obliquely to the laser propagation direction.

Back-electron video

Video corresponding to Fig. 4 (main paper), showing results of a 2D OSIRIS simulation of the emission of low-energy electrons opposite to the laser propagation direction.

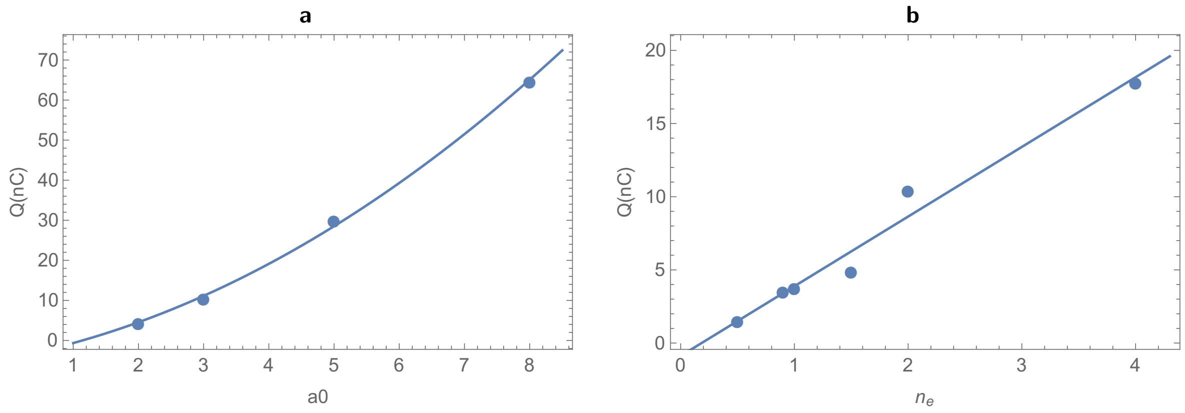

Figure S.8. Dependence of side-electron beam charge on a) laser a_0 and b) plasma density for laser $w_0 = 7\,\mu\text{m}$ and 0.5 mm propagation length.

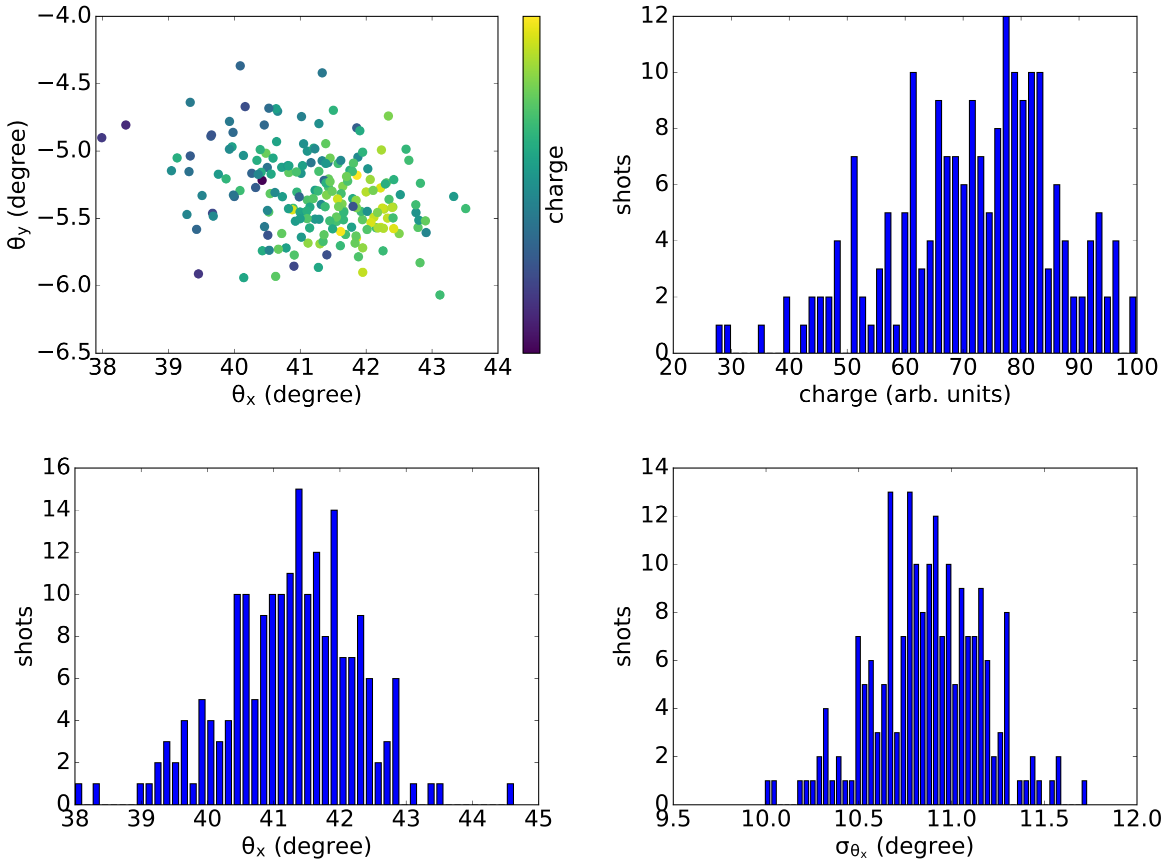

Figure S.9. Angular properties of oblique electron beams for 200 consecutive shots: mean pointing angle (top left), charge variation (top right), mean horizontal angle (bottom left) and mean horizontal divergence (bottom right).

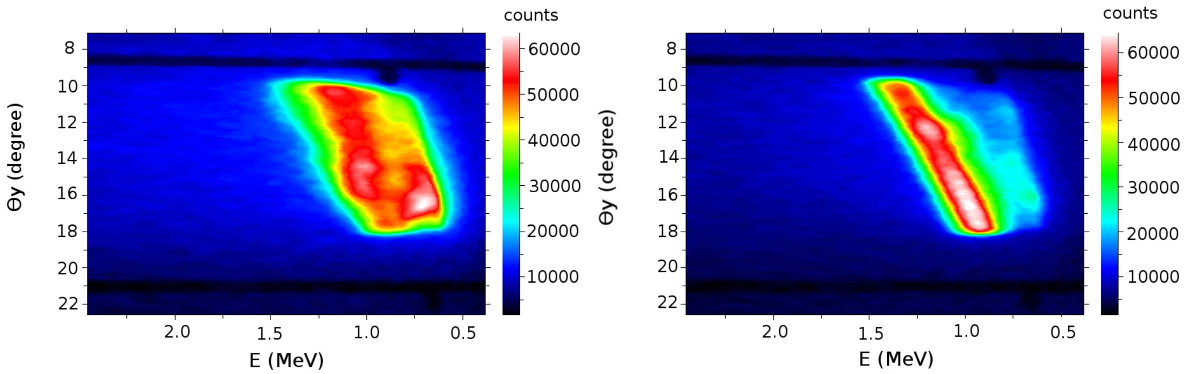

Figure S.10. Sample electron spectra of oblique electrons.

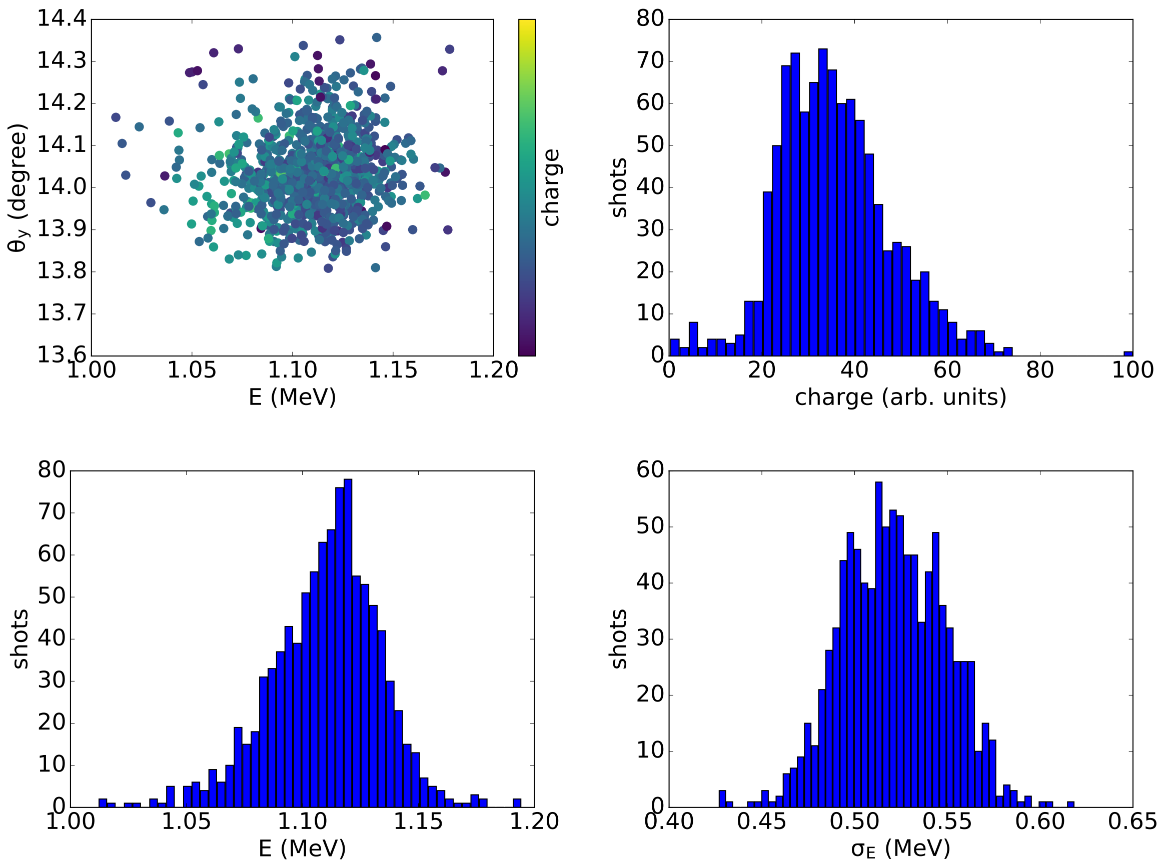

Figure S.11. Spectral properties of oblique electron beams for 5 runs containing a total of 984 shots: mean energy and vertical angle (top left), charge variation (top right), mean energy (bottom left) and mean energy spread (bottom right).

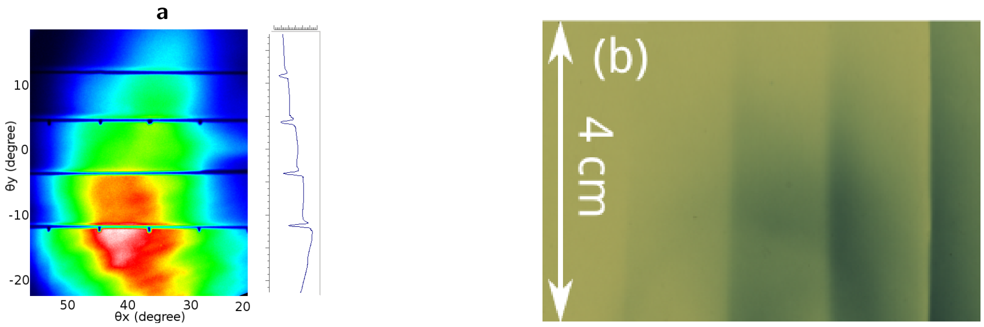

Figure S.12. a) Energy measurement of side-electrons on a Lanex screen behind a filter with 1 (bottom) to 5 (top) layers of 250 µm Al sheets. b) Energy measurement of backward electrons on a Gafchromic film behind a filter made of 1 (right) to 4 (left) layers of 50 µm Al sheets.

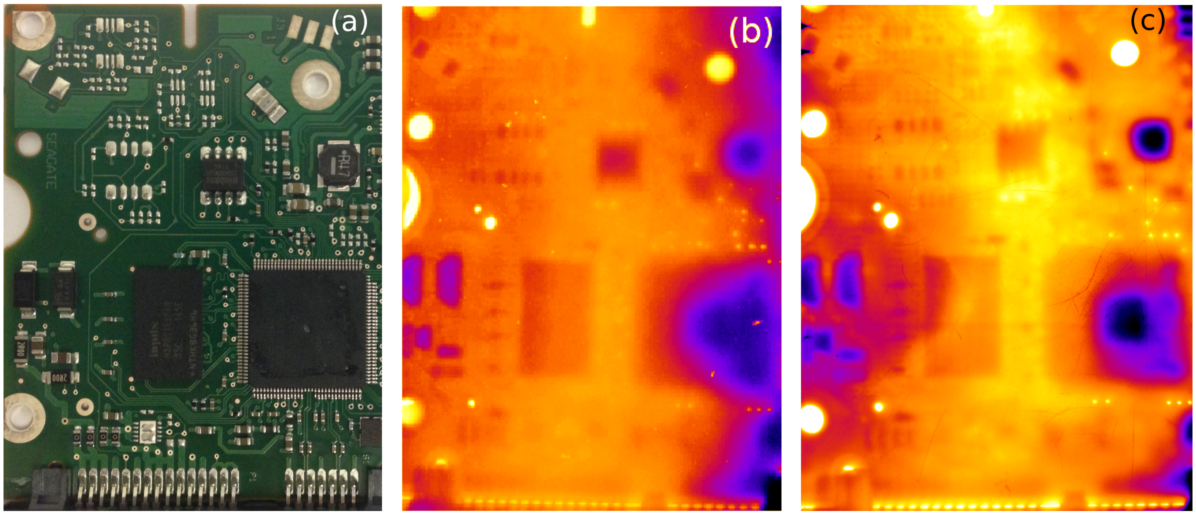

Figure S.13. Imaging measurements of a circuit board (a) using oblique electrons. (b) A Gafchromic film with 20 shots. (c) An image plate with a single shot.

References

1. Fonseca, R. *et al.* Osiris: A three-dimensional, fully relativistic particle in cell code for modeling plasma based accelerators. In *Computational Science-ICCS 2002, Pt III, Proceedings*, vol. 2331, 342–351 (2002).
2. Tsung, F. S. *et al.* Simulation of monoenergetic electron generation via laser wakefield accelerators for 5–25TW lasers. *Physics of Plasmas* **13**, 056708 (2006).
3. Bornatici, M. & Maj, O. Wave beam propagation in a weakly inhomogeneous isotropic medium: paraxial approximation and beyond. *Plasma Physics and Controlled Fusion* **45**, 707 (2003).
4. Pukhov, A. Strong field interaction of laser radiation. *Reports on Progress in Physics* **66**, 47 (2003).
5. Ivanov, M., Spanner, M. & Smirnova, O. Anatomy of strong field ionization. *J of Modern Optics* **52**, 165–184 (2005).
6. Hofmann, C., Landsman, A. S., Cirelli, C., Pfeiffer, A. N. & Keller, U. Comparison of different approaches to the longitudinal momentum spread after tunnel ionization. *J of Phys. B* **46**, 125601 (2013).
7. Pfeiffer, A. N. *et al.* Probing the longitudinal momentum spread of the electron wave packet at the tunnel exit. *Phys. Rev. Lett.* **109**, 083002 (2012).
8. Lemos, N. *et al.* Plasma expansion into a waveguide created by a linearly polarized femtosecond laser pulse. *Physics of Plasmas* **20** (2013).
9. Woolley, C. J., O'Keeffe, K., Chung, H. K. & Hooker, S. M. Time-resolved plasma temperature measurements in a laser-triggered hydrogen-filled capillary discharge waveguide. *Plasma Sources Science and Technology* **20**, 055014 (2011).
10. Kostyukov, I., Pukhov, A. & Kiselev, S. Phenomenological theory of laser-plasma interaction in "bubble" regime. *Physics of Plasmas* **11**, 5256–5264 (2004).
11. Lu, W. *et al.* Generating multi-gev electron bunches using single stage laser wakefield acceleration in a 3d nonlinear regime. *Phys. Rev. ST Accel. Beams* **10** (2007).
12. Jaroszynski, D. *et al.* Radiation sources based on laser–plasma interactions. *Philosophical Transactions of the Royal Society of London A: Mathematical, Physical and Engineering Sciences* **364**, 689–710 (2006).
